# Social evaluations of scientific occupations

**DOI:** 10.1038/s41598-022-23197-7

**Published:** 2022-10-31

**Authors:** Vukašin Gligorić, Gerben A. van Kleef, Bastiaan T. Rutjens

**Affiliations:** Department of Psychology, University of Amsterdam, Nieuwe Achtergracht 129B, 1018 WS Amsterdam, The Netherlands

**Keywords:** Psychology and behaviour, Psychology, Human behaviour

## Abstract

Science and scientists are among the key drivers of societal progress and technological developments. While research has demonstrated that science is perceived as heterogeneous, work on perceptions of scientists usually considers “scientists” as members of a homogeneous group. In the present research, we went beyond this general categorization by investigating differences in social evaluations of different types of scientists. Across four studies conducted in the UK and the US (total *N* = 1441), we discovered that members of the most frequently mentioned scientific occupations (35 and 36 respectively in each country) are seen as highly competent, relatively moral, but only moderately sociable. We also found that individuals perceive differences between scientific occupations across social dimensions, which were captured in clusters of scientific occupations. Chemists, biologists, and physicists represented the most mentioned and highly prototypical scientific occupations. Perceived prototypicality was primarily associated with competence ratings, meaning that, in the public’s view, to be a scientist means to be competent. Perceptions of morality and sociability varied notably across clusters. Overall, we demonstrate that focusing only on “scientists” leads to overgeneralization, and that distinguishing between different types of scientists provides a much-needed nuanced picture of social evaluations of scientists across occupations.

## Introduction

Anri Burton: “Knowledge is only valid when it's based on morality.”

Kris Kelvin: “Man is the one who renders science moral or immoral. Remember Hiroshima?”

(*Solaris*, 1972)

Contemporary societal problems, like the COVID-19 pandemic, have put both science and scientists in the spotlight. Scientific experts such as virologists and epidemiologists have increasingly been at the center of public attention, where they have been represented in various lights: from heroes who will stop the pandemic^1^ to cold and immoral profit-seeking agents^2^. Given that scientists are more and more present in the media, it is surprising how little research has investigated social evaluations of scientists. This dearth of research is even more notable when considering that “scientist” is not a monolithic category: various types of scientists can be identified even within the same scientific field (e.g., climatologists, meteorologists, and geophysicists investigating climate change). To the best of our knowledge, no research has systematically studied whether different scientific occupations elicit different evaluations, that is, whether people perceive meaningful differences between these occupational groups. In the present research, we attempted to fill this gap by investigating social evaluations of different types of scientists, and how these relate to perceptions of prototypicality. A comprehensive understanding of social perceptions of scientists would increase the understanding of previous studies (e.g., those focusing on the general category of “scientists”), as well as provide theoretical scaffolding for future studies by investigating whether the public sees scientists as a monolithic group, several groups, or even as individual scientific occupations. Finally, it would provide insights for potential interventions aimed at increasing public acceptance of science or the appeal of certain scientific careers.

What does a scientist look like in people’s minds? Early research showed that scientists are stereotyped as older, middle-aged males with beards and glasses, wearing white coats, and working in a laboratory^3,4^. Later, researchers developed a Draw-A-Scientist-Test which investigated stereotypes that children had by simply asking them to draw a scientist, and then counting how many of the seven stereotypical elements occur (e.g., lab coat, eyeglasses, facial hair)^5^. While this test went through several changes and improvements over time (e.g., by adding characteristics such as male sex, indications of danger, indoor work)^6^, findings seem to be relatively stable. As a recent review showed, the scientists’ image is not much different from the one found in classic research, so scientists are still stereotyped as white middle-aged or older males who wear lab coats and work indoors^7^. Notably, however, the once pervasive idea of a deranged and evil scientist who conducts dangerous experiments has become less prominent^7–9^.

While classic research focused on the scientist’s image, more recent research investigated the social evaluations they elicit, that is, perceptions of scientists’ psychological characteristics. This work is not independent of the previous line, given that the image of a scientist almost necessarily includes a perception of traits such as intelligence, brilliance, and/or genius^4^. Fiske and Dupree ^10^ investigated perceptions of different occupations and found that scientists and researchers are seen as highly competent, but only modestly warm^10^. Thus, scientists were classified into the cold-competent cluster that also included occupations such as lawyers and accountants. These findings were replicated and extended, showing that scientists are indeed perceived as highly competent and not very warm^11^. However, this research also found that the public perceives scientists as relatively moral. Therefore, although people view scientists as more competent than moral, they also see them as more moral than warm. Morality indeed plays an important role in the perception of scientists—even though scientists are seen as generally moral (honest, trustworthy), people still associate them with severely immoral behavior, particularly when the behavior involves moral purity violations^12^. Importantly, this second line of research utilizes social evaluation models, which we also employed in the present studies.

A fundamental issue in psychology concerns the number of dimensions along which individuals organize their social evaluations. The Stereotype Content Model (SCM)^13^ proposes that individuals first assess one’s *warmth* (whether someone has good or ill attention), and then *competence* (whether they are able to fulfill these intentions). More recent research suggests important differences between warmth and morality (e.g., politicians are often perceived as simultaneously friendly and cunning), even arguing for the primacy of the latter^14,15^. Addressing this issue, Ellemers and colleagues^16,17^ developed a three-dimensional model (the Behavioral Regulation Model; BRM) containing dimensions of *competence*, *sociability* (i.e., warmth in the SCM), and *morality*. Finally, the Dual Perspective Model (DPM)^18,19^ argues that both two superordinate factors—*agency* and *communion* (i.e., competence and warmth in the SCM)—could be divided into two facets: Communion consists of warmth and morality (mirroring the BRM), while agency includes competence (whether one is capable, intelligent) and assertiveness (whether one is confident).

Another social evaluation model is the ABC model of stereotypes^20^, which argues that three dimensions that emerge in spontaneous judgments are *agency* (referring more to structural position in a hierarchy, rather than competence in the sense above), *communion* (which subsumes both warmth and morality), and a very different dimension—*beliefs*. The last dimension is based along the continuum progressive-conservative, where groups that are more progressive challenge and change the status quo, whereas conservative groups prefer its maintenance. In the present research, we used the BRM, ABC, and DPM models to investigate scientists' perceptions. We used the BRM because it extends the SCM by emphasizing the role of morality. The ABC model was included for its innovative dimension of belief, which might be relevant for differentiating scientists (e.g., geneticists and nuclear physicists could challenge the status quo more than mathematicians). Finally, the DPM is relevant as its dimensional structure appears to be most appropriate according to the most recent integration of five different social evaluation models^21^.

Although the research described earlier provides insights into how people view scientists in general, it remains unclear to which specific occupation(s) the generic term “scientist” refers. Nevertheless, some of the characteristics described in classic research^34^ point to certain scientific occupations that might be seen as prototypical of scientists. For example, while lab coats and instruments are indicative of chemical occupations, equations are typical for physical and mathematical occupations. Seeing these occupations as prototypical of science also resonates well with the literature, as researchers used mathematicians^9^ or chemists^22^ as representatives of scientists. When asked about controversial science, biologists and chemists were found to be the most representative^23^. Similarly, physics, biology, and medicine are perceived as more scientific than sociology and economics^24^. In general, while some occupations are mentioned more often than others, no study has systematically investigated which occupations are most prototypical of science. This is problematic given the breadth and diversity of scientific occupations. Even more importantly, the homogenization of scientists can be misleading, given that both the structure of science and public evaluations of science are found to be heterogeneous^25^.

Science is by no means a uniform enterprise, neither from a philosophical point of view (e.g., the difference between natural and social sciences) nor from a psychological perspective. For example, science skepticism, defined as the systematic rejection of scientific method and evidence, has different levels across domains. Even though most people would agree that smoking causes cancer (98% in France)^26^, such consensus is much lower for other scientific issues^27^ such as anthropogenic effects on the climate (57% in the US)^28^ or safety of genetically modified (GM) food (13% across 20 countries)^29^. Similarly, the causes and correlates of science skepticism are heterogeneous as well, meaning that skepticism in different domains stems from different antecedents. For example, climate change skepticism is best predicted by political conservatism, evolution rejection is mostly dependent on religious views, while skepticism toward GM food is best predicted by science knowledge ^30,31^.

These various strands of research converge on the notion that science is a multifaceted concept. This notion should also apply to *scientists*. Even classic research showed that stereotypes between biologists, chemists, and physicists are somewhat different, so that biologists are closest to the American ideal, while physicists engender more negative stereotypes^3^. More recent research shows that publicly (vs. privately) funded scientists foster more positive social evaluations^11^ and trust^32^. Additionally, ideology affects the perceptions of different scientists in different ways. For example, conservatives (vs. liberals) prefer scientists working in production (industrial chemists, petroleum geologists) more than impact science (epidemiologists, oceanographers)^33^. Finally, people attribute different reasons for the success of individual scientists (e.g., Einstein succeeded because of his extraordinary talent, and Edison due to his efforts and perseverance), which has downstream consequences for people’s own motivation to succeed in science^34,35^. In sum, research indicates that not only science but scientists as well should not be simply viewed as one uniform group. However, none of the research discussed here has systematically investigated these differences.

The present research had two aims. The first aim was to systematically investigate social evaluations of an extensive list of different scientific occupations. We did this by mapping occupations on social evaluation dimensions and investigating whether meaningful clusters emerge. The second aim was to identify the most prototypical scientific occupations, and investigate which social evaluation dimensions contribute to this prototypicality.

To achieve these aims, we conducted four studies utilizing quota and representative sampling (see methods for details), two in the UK (Studies 1a and 1b), and two in the US (Studies 2a and 2b). In Study 1a, we utilized a bottom-up approach and asked participants to freely list scientific occupations that came to their minds. In Study 1b, we used this list to investigate social evaluations of these occupations using the BRM and ABC models of social evaluations, and how these evaluations relate to prototypicality perceptions. Study 2a was identical to Study 1a, as we wanted to use the same bottom-up approach but in another country (US). In Study 2b, we aimed to replicate Study 1b (which involved the BRM model) and extend it by using another model (the DPM). All materials, databases, analysis scripts, and supplemental information can be found at the Open Science Framework (OSF; link).

## Study 1a

The aims of Study 1a were to obtain a list of scientific occupations for the main study and to discover scientific occupations that are most accessible to participants.

### Results and discussion

Before analyzing the data, we took several steps in preprocessing to account for idiosyncrasies in responding (e.g., correcting spelling mistakes, capitalizing first letter entries, etc.). All of these steps are elaborated on in the Supplement. Based on the pre-processed list, we calculated the entry frequency for each scientific occupation. The final list contained 35 scientific occupations that had more than 15 counts (see the second column of Supplementary Table 1). As the table shows, the list is dominated by natural science occupations (nine out of the top ten). The most frequently mentioned occupations were *chemists*, *biologists*, and *physicists*. Interestingly, the list also includes several occupations that do not belong to natural sciences (e.g., *psychologists*, *archeologists*), with most of them in the lower part of the table (e.g., *anthropologists*, *sociologists*). Given that the list obtained in this study contained the most accessible and familiar occupations, we used it in Study 1b in which we investigated social evaluations of these occupations.

## Study 1b

This study aimed to map scientific occupations that were produced in Study 1a, using fundamental dimensions of social evaluations. This approach also allowed us to investigate whether meaningful clusters of scientific occupations emerge based on social evaluations, that is, whether or not all scientific occupations are perceived as belonging to the same category. Additionally, we wanted to see which occupations are seen as most prototypical of scientists, and which social evaluation dimensions contribute to this prototypicality.

### Results and discussion

#### Prototypicality and counts

We first investigated whether occupations’ prototypicality scores (also given in Supplementary Table 1) from Study 1b are correlated with counts from Study 1a. High correlations would indicate that the most prototypical occupations came first to people’s minds. On the other hand, low or no correlation would indicate that participants did not have the most prototypical occupations on their minds, suggesting that the list is not exclusive of less prototypical occupations, and thus representative to some extent. A correlation size of *r*(32) = 0.360, *p* = 0.036, suggests that latter is the case. This is even more evident when the correlation is calculated after leaving out the three occupations with the highest counts (chemist, biologist, physicist), *r*(30) = 0.178, *p* = 0.330.

#### Clusters of occupations

To test for clustering, we first had to calculate dimension ratings for each occupation. Since not every participant rated each occupation, we did not use the mean ratings, but wanted to control for idiosyncrasies^36^. We estimated means from a mixed model which included random intercept for participants (ICC_competence_ = 0.27, ICC_sociability_ = 0.39, ICC_morality_ = 0.44). Ratings for the BRM dimensions for each occupation are given in Supplementary Table 1. Means suggest that scientific occupations are judged to be highly competent (*M* = 5.88), moderately sociable (*M* = 4.61) and relatively moral (*M* = 5.20). Differences between all dimensions were significant, *t*s > 11.27 p_bonf_ < 0.001. Correlations between dimensions across occupations are presented in Table 1.

**Table 1:** Correlations between social evaluations and prototypicality.

	Competence	Sociability	Morality
**Competence**			
Sociability	−0.466**		
Morality	0.299^†^	0.624***	
Prototypicality	0.877***	−0.296^†^	0.374*

Correlations with prototypicality did not include generic occupations of “scientist” and “researcher”. ^†^*p* < 0.10, *p < 0.05, **p < 0.01, ***p < 0.001.

**Figure 1:**
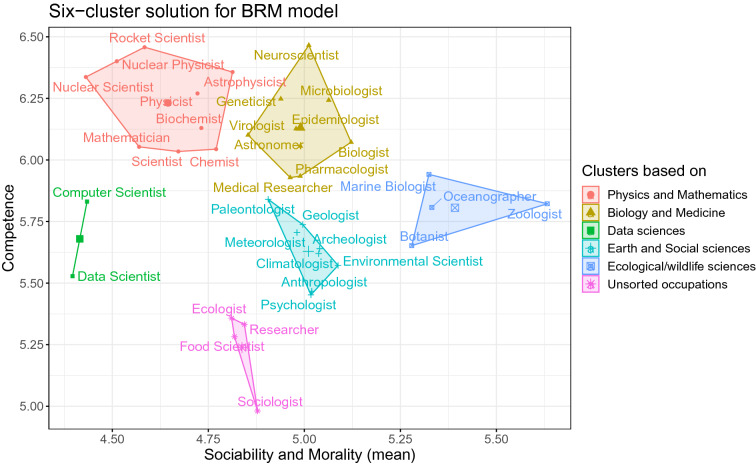
Six clusters of scientific occupations based on the ratings of competence, sociability, and morality. For the 2D representation, we collapsed sociability and morality because many models consider them subdimensions of a higher-order factor (named warmth^13^ or communion^19^). Lines represent cluster borders, with each cluster having its own color and symbol. Cluster centroids are represented with a large symbol in the cluster center.

To determine the number of clusters based on perceptions of competence, sociability, and morality, we first investigated the plot of within-cluster sums of squares using hierarchical clustering as a partitioning function. This indicated that any number of clusters between 2 and 6 is viable. Next, using the NbClust package in R (using Euclidian distance to compute dissimilarity matrix and Ward method as cluster analysis method), we investigated which of the two-to-six-cluster solution is most appropriate. The NbClust package uses 30 indices to determine which proposed number of clusters is the best according to the majority of indices. In the present study, NbClust showed that 10 indices suggested a 6-cluster solution. We performed cluster analysis using hierarchical clustering as an algorithm (Euclidian distance and Ward method). The 3D graph is not feasible to include in an article, which is why we present the six-cluster solution here. To present the clusters on a2D graph, wecollapsed sociability and morality into one dimension given that many models consider them to be subdimensions of a higher-order factor named warmth^13^ or communion^19^ (Fig. 1). We named the clusters according to the overlapping content of the respective occupations; however, in some cases, an occupation would better fit into a different cluster. Therefore, these names should be viewed as relatively arbitrary but nonetheless helpful in interpretation, rather than as reflecting the inherent structure of scientific occupations.

The figure shows several patterns. Most notably, the generic category “scientist” is found among physical and mathematical occupations, which are, together with biomedical occupations, rated as the most competent. It is also noteworthy that there is an overlap between these occupations, as illustrated by the membership of “astronomer” which was classified as a biomedical occupation. Another two clearly defined clusters are occupations concerned with wildlife (most sociable and moral) and those concerned with computer science (averagely competent and less sociable relative to other occupations). The cluster in the center of the graph covers the earth and social sciences (average on all dimensions), while the final cluster contains occupations that do not seem to have anything in common content-wise, but are seen as less competent compared to other occupations. Interestingly, it also contains the term “researcher”, suggesting its different perception from the “scientist” which has a higher competence score. Importantly, almost identical results are obtained when we used *k-*means clustering to determine cluster membership, with the only difference being “astrophysicist” which is classified as a biomedical occupation (supporting the notion of conceptual overlap between two clusters). We also probed the stability of the 6-cluster solution by using different outlier strategy and calculation of dimensions scores. This returned very similar results, indicating the stability of the findings.

#### Social evaluation dimensions and prototypicality

We next investigated which social dimensions contribute to prototypicality perceptions. Rating an occupation as more prototypical of scientists was associated with perceptions of higher competence, lower sociability, and higher morality (Table 1). Next, we included three social evaluation dimensions in linear regression as predictors of prototypicality. The model was significant, *F*(3,30) = 37.190, *p* < 0.001, *adj R*^2^ = 0.77, showing that only higher levels of competence contributed to the perception of prototypicality, *t* = 5.015, *p* < 0.001, with no effects of sociability or morality (*t*s < 0.389, *p*s > 0.70).

In sum, Study 1b showed that scientists are perceived as highly competent, relatively moral, and moderately sociable. Yet, people distinguish between different types of scientists along these social dimensions, which results in six clusters of scientific occupations that vary in the extent to which they are perceived as competent, sociable, and moral. Finally, the most prototypical occupations are those rated as the most competent.

## Study 2a

Our list of scientific occupations obtained in Study 1a was based on a UK sample, leaving a possibility the list is context-dependent (e.g., on a sample and/or country characteristics). In Study 2a, we aimed to test to what extent a different sample from a different country would produce a similar list of occupations. Results were highly similar to those obtained in the UK: Among occupations with 15 and more counts (36 in total), all occupations from the UK list, except “pharmacologist”, were present in the US list. On the other hand, two additional occupations (“statistician” and “hydrologist”) were featured in the list generated by the U.S. participants (Supplementary Table 1). This overlap suggests that the list is generalizable with occupations of *chemist*, *biologist*, and *physicist* again forming the top three (see OSF for tables from Studies 1a and 2a where occupations are sorted by counts of each study). We used this list to investigate social evaluations of scientists in Study 2b.

## Study 2b

Study 2b had the same aims as Study 1b. However, instead of the ABC model (which showed poor reliability), we used the DPM, which includes a competence-assertiveness distinction (as compared to a single competence dimension in the BRM model). Besides mapping social evaluations of different scientific occupations, we again examined which dimensions contribute to prototypicality.

### Results and discussion

#### Prototypicality and counts

As in the first study, prototypicality scores from Study 2a had low correlations with counts from Study 2b, *r*(33) = 0.236 and −0.038, *p*s > 0.17 (respective correlation sizes when all occupations are included and when the three occupations with the highest counts are left out). Again, these indicate that the list is not exclusive of less prototypical occupations, and thus representative to some extent.

#### BRM model

We fully report the results for the BRM model in the Supplement. In short, we replicated findings from Study 1b: We discovered the same clusters, while only competence ratings contributed to prototypicality. We next wanted to see whether clustering changes when a different model of social evaluations is used. Additionally, given that the DPM model distinguishes between two facets of competence, we could investigate whether effects on prototypicality involve competence (i.e., ability, capability) and/or assertiveness (i.e., ambition, confidence). Below, we summarize the results of the analyses on the DPM model dimensions (see the supplement for full results).

#### DPM model

**Figure 2:**
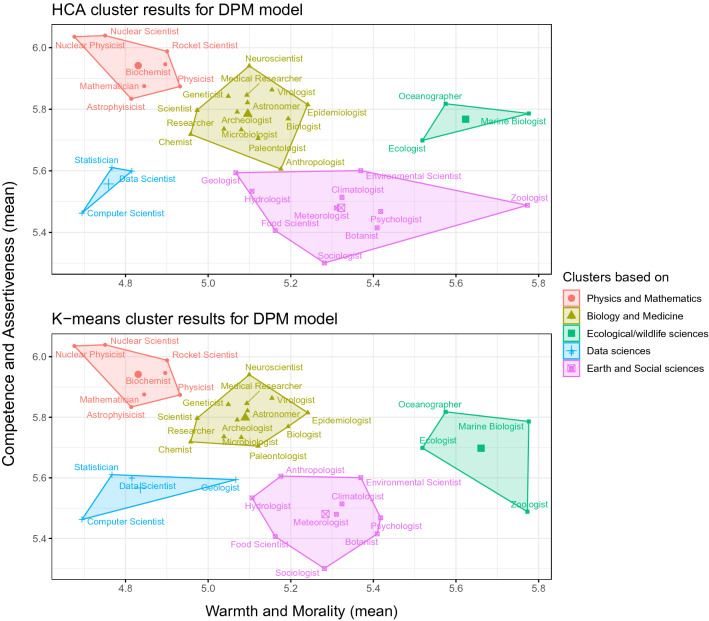
Five-cluster solution in Study 2b when classification is conducted using Hierarchical clustering (top) and K-means clustering (bottom). To present the clusters on a 2D graph, we calculated means of competence and assertiveness (subdimensions of agency), and warmth and morality (subdimensions of communion)^19^.

We used the same approach in determining the number of clusters as in Study 2. NbClust showed that most indices (8) suggested the 5-cluster solution. Cluster memberships for this solution based on HCA and K-means are given in Fig. 2. Apart from several idiosyncrasies such as “incorrect” classification (in terms of cluster content) of “zoologist” and “anthropologist” in HCA, and “geologist” in *K*-means, there is another important thing to note. Cluster analysis based on the BRM and DPM yielded very similar clusters in terms of their content. The only major difference is that the DPM had a 5-, instead of a 6-cluster solution. This occurred due to the merging of the BRM’s unsorted occupations with earth and social science occupations, which is in line with the cluster’s content. When a 5-cluster solution is imposed on the BRM model, unsorted occupations merge with earth and social science occupations, which is in line both with the cluster’s content and results from the DPM model. On the other hand, imposing a 6-cluster solution on the DPM model does not provide the 6-cluster solution of the BRM model, suggesting its low stability across models. Overall, with some differences depending on the method and social dimensions model used, we believe that these data, as well as their interpretation, point to a five-cluster solution (Physics and Mathematics, Biology and Medicine, Data sciences, Earth and Social sciences, Ecological/wildlife sciences).

#### Social evaluation dimensions (DPM) and prototypicality

We next investigated which social dimensions contribute to the perception of prototypicality when included in one model. Linear regression with four social evaluations as predictors showed that the model was significant, *F*(4, 30) = 20.45, *p* < 0.001, *adj R*^2^ = 0.70. Higher levels of competence contributed to prototypicality ratings (*t* = 3.612, *p* < 0.01), while warmth (*t* = 1.231, *p* = 0.23), morality (*t* = 0.111, *p* = 0.912), and assertiveness did not (*t* = 0.868, *p* = 0.39).

## General discussion

In the present research, we systematically investigated social evaluations of scientists and their consequences for perceived prototypicality. We found that scientists working in the most frequently mentioned scientific occupations are generally seen as highly competent, relatively moral, and only moderately sociable. However, we also discovered different clusters of scientific occupations, based on participants’ perceptions in terms of social evaluations. The defining characteristic of scientists is perceived to be competence (ability), as this was the only social evaluation dimension that contributed to prototypicality. Highly prototypical scientific occupations that most commonly come to mind are chemists, biologists, and physicists, making them the most representative occupations in science. However, different clusters of scientific occupations also varied notably in perceptions of sociability and morality. This variation could have consequences for science communication. Consider a data scientist and a social scientist communicating climate science to the public. Whereas the data scientist might benefit from a message frame that bolsters their perceived warmth, a message frame that stresses competence might help the social scientist more. By building on various social evaluation models, the current findings provide a theoretically informed way to understand the *heterogeneity of scientists* and use this understanding to improve science communication.

In general, our results are in line with previous research which found that scientists are seen as highly competent, relatively moral, but only moderately warm ^10,11^. The key contribution of the current work is that people make meaningful differences between different types of scientists. This is in line not only with the findings on the heterogeneity of science^25^, but also with research showing that different types of scientists elicit different perceptions^32,35^. One important contribution of our approach is that we systematically compared multiple occupational groups of scientists, allowing for a detailed study of this heterogeneity.

An obvious question that arises concerns the number of clusters that can be formed. While we discovered five of them, we believe that there is no single answer to this question, for several reasons. Firstly, the number probably depends on social evaluation models, as evidenced by somewhat different cluster solutions when the BRM vs. DPM model is used. Secondly, it also likely depends on the country (or sample) where ratings are generated, so results might be different in more dissimilar countries (socio-politically or culturally). Although our findings from UK and US largely converged, this is not surprising given the similarities between these two countries. After all, it is the Western culture (e.g., literature and movies), from where the idea of the brilliant, mad scientists emerged^8^. Finally, cluster analyses can be subjective, so the number of clusters may partially depend on analytical decisions. In sum, our findings are mainly descriptive, and the consequences and causes of these social evaluations are yet to be studied.

If there are differences between different scientific occupations, what do people think of when asked about “scientists”? Our research suggests that the general public thinks about the physical and biomedical sciences, which is a cluster that was found to always include the general term “scientist”. Scientists working in the areas of earth, social, and data sciences were seen as somewhat less competent than the prototypical group of occupations. On the other hand, ecological/wildlife sciences are seen as too sociable and moral to be within the strictly scientific group. Within the physical and biomedical sciences, three occupations specifically—physicists, chemists, and biologists—were most often mentioned as examples of scientists. This is in line with much of the previous work on scientists' evaluations and stereotypes^3,6,23^, but it also illustrates that employing other occupations as substitutions for the general term “scientists” might be less appropriate (e.g., mathematicians)^9^.

The current research is not without limitations. Firstly, our samples of scientific occupations might not be exhaustive because they included a limited number of occupations. Secondly, we did not ask participants if they knew the occupations they were rating in Studies 1b and 2b. However, occupation lists, which were highly similar in the UK and the US, were obtained using a bottom-up approach which ensured that the occupations we included are recognizable as they were repeatedly mentioned. This also underscores the trade-off between the exhaustiveness (number) and familiarity of added occupations: Many scientific occupations that, in principle, could be added might be unknown to the general public (e.g., herpetologists). In addition, while one general occupation could hypothetically be split up into many more specific occupations, the difference between them will be perceived only if people are (very) familiar with the occupations. Our studies showed this was the case for prominent scientific occupations (e.g., two subordinate categories of physicists, nuclear- and astro- were also included), but not others (e.g., psychologists were only mentioned as a general occupation).

Whereas we focused on social evaluations as one aspect of perceptions of scientists, future research could employ other types of perceptions of scientists (e.g., stereotypes relating to their gender, age, or ethnicity^5–7^) or other traits that are not necessarily part of the social evaluation models (e.g., masculinity or creativity) and investigate which characteristics are most prominently associated with prototypicality, complementing our findings. Another possible line of research for future studies concerns the causes of social evaluations of scientists. A plausible candidate would be the (mis)match between actual competencies and social evaluations, as the former could be causing the latter (for example, see ^36^). It is also conceivable that social evaluations of scientists only partially overlap with actual competencies, however, in which case subsequent investigations could seek to uncover alternative sources of such evaluations. Lastly, social evaluations are likely to shape trust in scientists. As suggested, a perceived lack of warmth (which encompasses sociability and morality) contributes to lower trust^10^. Future research should investigate the relative impact of these perceptions (and their interplay) on trust in scientists. Furthermore, taking into account the current findings, this should be done across different scientific occupations. More specifically, distrust towards various occupations may stem from various aspects of social perceptions. For instance, it could be that distrust of virologists is mainly due to a perceived lack of competence (“they don’t know anything about the virus”), while distrust in geneticists may be shaped primarily by a perceived lack of morality (“they are playing God”)^37^. In short, disentangling the effects of different social evaluations on trust in different types of scientists is an important future research direction.

## Conclusion

Scientists—an umbrella term that, for the general public, primarily refers to chemists, biologists, and physicists—foster positive perceptions in general, with competence standing out as their most prototypical trait. Although at first blush these positive evaluations of scientists are good news for scientists and science advocates, the current data also show that these perceptions are not uniform, as people differentially evaluate different scientific occupations. Although the causes and consequences of these different evaluations are still unexplored, they demonstrate that “scientists” are not a homogenous group, at least in the public eye. Acknowledging the complexity of social evaluations of scientists is an important step in better understanding perceptions of scientists and science, and how these shape public acceptance of science.

## Method

### Method Study 1a

#### Participants

We recruited 300 UK participants using Prolific, pre-screening only participants with an approval rate of 100%. We used quota sampling, based on the demographic quotas (age and gender) from the Office for National Statistics (ONS, 2011). Our sample was balanced on gender (149 males, 149 females, 2 indicated “other”) with a mean age of 42.8 years (*SD* = 14.5). Two percent of the sample had education less than high school, 31% had completed high school, 8% were students, 39% had an undergraduate degree, and 21% had a graduate degree (percentages total over 100 due to rounding). Participants were paid £0.88 (around €1 or $1.20) for their participation of approximately seven minutes.

#### Procedure

In this study, we utilized a bottom-up approach and asked participants to write down all scientific occupations that came to their minds during five minutes. We preferred this approach over utilizing an existing list for several reasons. First, existing lists of scientific occupations differ notably from one another as they were compiled based on divergent inclusion criteria. Rather than arbitrarily selecting one such list, which might introduce bias in our investigation, we decided to adopt a bottom-up approach to generate our own list. Second, we were interested in how the public sees scientists. Therefore, we sought to include only scientific disciplines that participants can spontaneously think of. This approach ensured that the occupations that ended up on our list are familiar to participants, so that rating them on various social evaluation dimensions is a meaningful task. As an added benefit, by using a bottom-up approach we obtained counts that allowed us to discover the three most salient occupations.

Participants had 25 boxes they could fill out. However, we neither set a minimum nor maximum of occupations they had to name, so as not to encourage consulting other sources of information. The number of boxes was based on a small pilot in which three psychology researchers managed to fill out around 20 occupations in five minutes. Therefore, participants had only one task of coming up with scientific occupations. After signing the consent form, they were presented a page instructing them to write down all scientific occupations they could think of, without consulting any other sources of information such as the Internet. Next, participants were presented with the boxes and the 5-min timer. When 5 min passed, the page automatically closed. On the final page, participants were thanked and redirected to the submission page.

### Method Study 1b

#### Participants

We recruited 482 UK participants using Prolific, allowing only participants with an approval rate of 95% to take part in the study. This number of participants was based on a calculation that each occupation should have 80 ratings (each participant rated six occupations, see procedure). Same as in the previous study, we used quota sampling, based on the demographic quotas. Participants were paid £1.50 (around €1.75 or $2) for their participation of approximately eleven minutes.

Before data analysis, we excluded inattentive respondents (*n* = 4) and speeders, that is, respondents whose completion time was twice faster than the median of 562 s (*n* = 9). We also excluded 58 more participants as outliers (see Outliers calculation). This resulted in the final sample of 411 participants (206 males, 204 females, 1 indicated “other”), with a mean age of 43.3 (SD = 14.2). One percent of the sample had education less than high school, 33% had completed high school, 7% were students, 40% had an undergraduate degree, and 19% had a graduate degree.

#### Outliers calculation

Regarding the outliers, we first excluded univariate outliers on the prototypicality measure for each occupation using median absolute deviation (MAD). Therefore, we first tested outliers for prototypicality scores for chemists (*n* = 4 based on the cut-off of 35), then biologists (*n* = 1 based on the cut-off of 30), and so on for each of the 34 occupations that had prototypicality ratings. This resulted in the exclusion of 50 participants. Next, we tested for multivariate outliers (Mahalanobis distance) on each social evaluation item (25 in total) for each occupation. That is, we checked for multivariate outliers in responding to the perceptions of “scientist”, then “researcher”, and so on. Based on this strategy, we excluded eight more participants.

#### Materials and procedure

##### Occupations

Our final list used for Study 1b included 36 occupations (see Supplementary Table 1). In addition to the list with which participants from Study 1a would come up, we also planned to use generic terms of “scientist” and “researcher”. However, since the list from Study 1a already contained the term “researcher”, we only added the term “scientist” to the 35-occupations list, resulting in 36 occupations to be rated. To prevent fatigue, each participant was presented with six randomly selected occupations and asked to rate them on the following measures.

##### Measures

After reporting their demographics, participants were presented with six scientific occupations which they rated on two groups of social evaluation measures (based on the BRM and ABC models) and prototypicality. The presentation order of the group of social evaluation measures was randomized. After completing all measures, participants were asked to honestly indicate how attentive they were, with a note that it would not affect their payment, only our analyses (1 = *not at all attentive* to 5 = *extremely attentive*).

##### Competence, sociability, and morality

All three fundamental dimensions from the BRM model were measured using three items. Participants answered to what extent they felt that an average [scientific occupation] was “competent”, “intelligent”, “skilled” (competence), “likable”, “warm”, “friendly” (sociability), and “honest”, “sincere”, “trustworthy” (morality)^15^. All nine items were presented in random order. Answers were given on a seven-point Likert scale (1 = *not at all* to 7 = *extremely*). The scores for each dimension were calculated by taking the mean ratings of three respective items.

##### Agency, beliefs, and communion

To measure the dimensions of the ABC model, we used a seven-point bipolar scale (−3 to 3), with the following anchors for agency: “powerless-powerful”, “dominated-dominating”, “low status-high status”, “poor-wealthy”, “unconfident-confident”, “unassertive-competitive”. Beliefs were measured with the adjectives “religious-science oriented”, “conventional-alternative”, “conservative-liberal”. Communion was measured using the anchors “untrustworthy-trustworthy”, “dishonest-sincere”, “repellent-likable”, “threatening-benevolent”, “cold-warm”, “egoistic-altruistic”.

##### Prototypicality

To measure the prototypicality of each occupation, we asked participants to rate to what extent members of a given scientific occupation were good examples of scientists. Participants answered using the slider from 0 = *not at all*, to 100 = *extremely*. The question of prototypicality was not presented for generic occupational terms of “scientist” and “researcher”, and was always presented after the social evaluation questions.

##### Reliabilities

Since each occupation had its own assessments of social dimensions, there were 36 reliability scores for each of the social dimensions measured above (216 scores in total). As for the BRM model, all measures showed good reliabilities with median Cronbach’s alpha values of α = 0.88 for competence (range 0.77–0.94), α = 0.92 for sociability (range 0.87–0.95), and α = 0.91 for morality (range 0.79–0.95). On the other hand, reliabilities of ABC measures were slightly lower: Agency’s median α = 0.83 (range 0.72–0.90), communion’s median α = 0.83 (range 0.65–0.91), while the reliability of beliefs measure was poor, median α = 0.55 (range 0.23–0.79). Given the low reliability of the beliefs scale, using this measure for analyses would not be valid, which is why we do not report analyses based on the ABC model.

### Method Study 2a

Using Prolific and its representative sample feature, we recruited 303 participants in the US (147 males, 153 females, 3 indicated “other”; *M*_age_ = 44.9, *SD* = 16.6). Less than one percent of participants had education lower than high school (0.3%), 31% completed high school, 13% were students, 31% had an undergraduate degree, and 25% had a graduate degree. The procedure was identical to Study 1a, and data pre-processing and processing followed the same logic (see Supplement).

### Method Study 2b

#### Participants

We recruited 495 US participants using Prolific’s representative sample feature. Participants were paid £1.80 (around €2.15 or $2.40) for their participation of approximately twelve minutes. Before data analysis, we excluded four inattentive respondents and 15 speeders (completion time twice faster than the median 722 s). We also excluded 49 more participants as outliers (see Outliers calculation). This resulted in the final sample of 427 participants (225 males, 198 females, 4 indicated “other”), with a mean age of 45.9 (SD = 16.3). One percent of the sample had education less than high school, 27% had completed high school, 10% were students, 36% had an undergraduate degree, and 26% had a graduate degree.

#### Outliers calculation

We calculated the number of outliers using the same outlier strategy as in Study 1b (MAD for univariate outliers on prototypicality and Mahalanobis distance for all other items). However, MAD returned 89 participants as outliers, which we deemed too big a proportion of the sample. For this reason, we switched to using three standard deviations from the mean as a univariate outlier strategy (excluding 43 participants), and Mahalanobis distance for multivariate outliers on each social evaluation item, as well as variables of trust and distance (excluding 6 participants). No data analyses were performed before outlier exclusion.

#### Materials and procedure

We used the same approach as in Study 1b. That is, we added “scientist” and “researcher” to the list, and had participants rate only six randomly selected occupations. The same measures of the BRM model and the prototypicality were used. New measures included the DPM model dimensions (we also measured psychological distance and trust toward each occupation; however, this was part of another research project, and therefore we do not report results for these variables here).

##### Competence, Assertiveness, Warmth, Morality

To measure the dimensions of the DPM model, we used a seven-point bipolar scale (-3 to 3), with opposite anchors for each dimension^19,38^. Each dimension was measured using five items. To measure competence, we used anchors such as “incapable-capable”, and assertiveness was measured using pairs like “unconfident-confident”. Warmth was measured using anchors such as “unfriendly-friendly”, while morality had pairs like “unjust-just”. The scores were calculated as means of items belonging to the respective dimensions.

##### Reliabilities

For each of the social dimensions above, there were 37 reliability scores (259 scores in total). As for the BRM model, all measures showed good reliabilities with median Cronbach’s alpha values of α = 0.89 for competence (range 0.69–0.95), α = 0.92 for sociability (range 0.86–0.96), and α = 0.89 for morality (range 0.78–0.93). Reliabilities of DPM measures were similar: competence’s median α = 0.91 (range 0.78–0.95), assertiveness’ median α = 0.87 (range 0.72–0.93), warmth’s median α = 0.92 (range 0.88–0.95), and morality’s median α = 0.88 (range 0.77–0.93).

### Ethics approval statement

Ethical approval was granted by the Institutional Review Board of the Department of Psychology at the University of Amsterdam. All studies were conducted in accordance with relevant guidelines and regulations of the institution. Informed consent was obtained from all participants.

## Supplementary Information

Supplementary Information.

## Data Availability

All data and codes are available at Open Science Framework: https://osf.io/cpjyd/?view_only=9f8ae089d0a34c1e9a8d9ea048121c5f.
