# Supplementary material for: Social evaluations of scientific occupations
Source: Sci Rep. 2022 Oct 31;12:18339. doi: 10.1038/s41598-022-23197-7 (PMC9622917; doi:10.1038/s41598-022-23197-7)
Supplement: Supplementary file 1 — Supplementary Information. [file 41598_2022_23197_MOESM1_ESM.docx]

**Social evaluations of scientific occupations**

Vukašin Gligorić*, Gerben A. van Kleef, Bastiaan T. Rutjens

Department of Psychology, University of Amsterdam

***Corresponding author:** Vukašin Gligorić,

Department of Psychology, University of Amsterdam, Nieuwe Achtergracht 129B 1018 WS Amsterdam, The Netherlands.

ORCID: 0000-0001-7528-6806

Email: [v.gligoric@uva.nl](mailto:v.gligoric@uva.nl)

**Steps in Study 1a**

1. Manually correct spelling mistakes in Excel (“Physisist” -> “Physicist”)
2. Import database
3. Capitalize first letter (“chemist” -> “Chemist”)
4. Remove spaces before and after (“Chemist “ -> “Chemist”)
5. Remove ‘s’ at the end of occupations (“Chemists” -> “Chemist”)
6. Count the entries (934 unique entries/occupations)
7. Selecting only occupations that have counts over two (187 entries)
8. Renaming occupations:
   1. Psychologist (Psychology)
   2. Pharmacologist (Pharmaceutical Researcher)
   3. Chemist (Chemical Scientist, Chemistry, Chemistry Researcher)
   4. Physicist (Physics)
   5. Geneticist (Genetic Scientist)
   6. Environmental Scientist (Environmentalist, Environmental)
   7. Climatologist (Climate Scientist)
   8. Microbiologist (Micro biologist)
   9. Biologists (Biology, Biological)
   10. Astronomer (Astronomist, Astronomy)
   11. Researcher (Research, Scientific Researcher)
   12. Volcanologist (Vulcanologist)
   13. Biochemist (Bio Chemist)
   14. Food Scientist (Food)
   15. Archeologist (Archaeologist)
   16. Medical Researcher (Medical, Medical Scientist, Medical Research, Medicine)
   17. Computer Scientist (Computer Science)
   18. Nuclear Scientist (Nuclear)
9. Cut-off (arbitrary in previous studies): 5% i.e., 15 counts. In total, 57 occupations
10. Delete non-scientific occupations (second column represents counts):

| Doctor | 98 |
| --- | --- |
| Pharmacist | 93 |
| Engineer | 54 |
| Science Teacher | 51 |
| Forensic Scientist | 50 |
| Astronaut | 44 |
| Chemical Engineer | 39 |
| Vet | 39 |
| Pathologist | 38 |
| Dentist | 35 |
| Lab Technician | 31 |
| Nurse | 29 |
| Psychiatrist | 24 |
| Surgeon | 23 |
| Teacher | 19 |
| Radiologist | 18 |
| Inventor | 17 |
| Laboratory Technician | 17 |
| Phlebotomist | 17 |
| Physician | 16 |
| Neurologist | 15 |
| Research Scientist | 15 |

1. Number of occupations: **35**
2. When no pre-processing is done (from step 4 on), a similar list is obtained

**Supplementary Table 1.**

Supplementary Table 1. Counts, prototypicality, and social evaluations (BRM) ratings for different scientific occupations in all studies

|  | Studies 1a & 1b (UK) | | | | | Studies 2a & 2b (US) | | | | |
| --- | --- | --- | --- | --- | --- | --- | --- | --- | --- | --- |
|  | Counts | PROTO | COMP | SOC | MOR | Counts | PROTO | COMP | SOC | MOR |
| Chemist | 229 | 85.13 | 6.04 | 4.39 | 5.15 | 214 | 91.93 | 6.27 | 4.08 | 5.23 |
| Biologist | 193 | 85.42 | 6.07 | 4.83 | 5.41 | 206 | 91.34 | 6.11 | 4.63 | 5.28 |
| Physicist | 175 | 86.30 | 6.27 | 4.22 | 5.22 | 159 | 89.37 | 6.24 | 4.10 | 5.22 |
| Geologist | 93 | 77.17 | 5.74 | 4.75 | 5.24 | 145 | 82.00 | 5.96 | 4.45 | 5.36 |
| Astronomer | 89 | 78.66 | 6.06 | 4.71 | 5.27 | 126 | 82.22 | 6.06 | 4.36 | 5.31 |
| Marine Biologist | 79 | 81.93 | 5.94 | 5.17 | 5.47 | 72 | 86.42 | 6.08 | 5.33 | 5.50 |
| Astrophysicist | 74 | 86.57 | 6.36 | 4.36 | 5.27 | 55 | 90.41 | 6.32 | 4.09 | 5.23 |
| Psychologist | 72 | 63.42 | 5.45 | 4.86 | 5.18 | 72 | 64.07 | 5.44 | 4.84 | 4.94 |
| Microbiologist | 68 | 86.84 | 6.24 | 4.65 | 5.48 | 54 | 88.06 | 6.21 | 4.21 | 5.15 |
| Zoologist | 65 | 75.91 | 5.82 | 5.60 | 5.66 | 72 | 82.55 | 5.76 | 5.50 | 5.43 |
| Botanist | 62 | 77.96 | 5.65 | 5.23 | 5.33 | 93 | 80.11 | 5.82 | 5.08 | 5.43 |
| Biochemist | 58 | 84.17 | 6.13 | 4.36 | 5.10 | 41 | 92.20 | 6.31 | 4.03 | 5.13 |
| Meteorologist | 58 | 73.57 | 5.71 | 4.81 | 5.15 | 67 | 76.17 | 5.43 | 5.22 | 5.12 |
| Virologist | 53 | 85.58 | 6.10 | 4.45 | 5.26 | 36 | 89.70 | 6.06 | 4.28 | 5.39 |
| Archaeologist | 49 | 66.65 | 5.64 | 4.84 | 5.24 | 101 | 82.21 | 5.97 | 4.44 | 5.10 |
| Mathematician | 42 | 71.91 | 6.05 | 4.09 | 5.05 | 53 | 78.72 | 6.29 | 3.99 | 5.18 |
| Medical Researcher | 41 | 78.26 | 5.93 | 4.60 | 5.33 | 40 | 86.45 | 6.09 | 4.37 | 5.30 |
| Geneticist | 40 | 84.31 | 6.25 | 4.63 | 5.25 | 38 | 89.14 | 6.15 | 4.28 | 5.14 |
| Paleontologist | 37 | 75.84 | 5.84 | 4.63 | 5.19 | 43 | 82.55 | 5.91 | 4.63 | 5.29 |
| Ecologist | 35 | 73.84 | 5.36 | 4.65 | 4.98 | 31 | 83.92 | 5.71 | 5.03 | 5.47 |
| Environmental Scientist | 33 | 75.87 | 5.57 | 4.92 | 5.26 | 48 | 84.13 | 5.71 | 4.80 | 5.24 |
| Nuclear Physicist | 32 | 87.09 | 6.40 | 4.03 | 5.00 | 23 | 93.03 | 6.48 | 3.80 | 4.95 |
| Epidemiologist | 29 | 78.15 | 6.13 | 4.57 | 5.39 | 28 | 82.85 | 5.99 | 4.40 | 5.25 |
| Computer Scientist | 28 | 70.42 | 5.83 | 4.09 | 4.78 | 36 | 69.25 | 5.94 | 3.86 | 4.8 |
| Nuclear Scientist | 24 | 85.97 | 6.34 | 3.97 | 4.89 | 20 | 90.53 | 6.40 | 3.83 | 5.02 |
| Rocket Scientist | 24 | 84.58 | 6.46 | 4.14 | 5.02 | 24 | 91.35 | 6.36 | 4.17 | 5.16 |
| Anthropologist | 22 | 71.32 | 5.47 | 4.89 | 5.14 | 60 | 77.97 | 5.76 | 4.60 | 5.11 |
| Oceanographer | 20 | 73.97 | 5.81 | 5.15 | 5.51 | 40 | 86.29 | 6.08 | 5.01 | 5.40 |
| Climatologist | 18 | 76.68 | 5.62 | 4.76 | 5.31 | 22 | 78.08 | 5.60 | 4.64 | 5.22 |
| Data Scientist | 17 | 68.07 | 5.53 | 4.04 | 4.76 | 18 | 73.57 | 5.81 | 4.07 | 4.93 |
| Food Scientist | 16 | 68.54 | 5.28 | 4.68 | 4.96 | 16 | 71.78 | 5.53 | 4.74 | 5.06 |
| Neuroscientist | 16 | 87.98 | 6.47 | 4.48 | 5.55 | 34 | 92.37 | 6.32 | 4.24 | 5.43 |
| Sociologist | 16 | 58.93 | 4.98 | 4.89 | 4.86 | 31 | 65.22 | 5.23 | 4.76 | 4.85 |
| Pharmacologist | 18 | 80.31 | 5.93 | 4.59 | 5.39 | / | / | / | / | / |
| Statistician | / | / | / | / | / | 21 | 71.40 | 5.82 | 3.96 | 5.03 |
| Hydrologist | / | / | / | / | / | 19 | 82.20 | 5.72 | 4.50 | 5.16 |
| Researcher | 51 | / | 5.33 | 4.67 | 5.02 | 43 | / | 5.72 | 4.35 | 5.06 |
| Scientist | / | / | 6.03 | 4.26 | 5.09 | / | / | 6.12 | 4.06 | 5.13 |

*Note*. Occupations are sorted by counts from Study 1a. “Pharmacologist”, “statistician”, and “hydrologist” are presented after occupations that were used in both countries, and have values only for one country. “Researcher” and “Scientist” are generic terms and did not contain prototypicality questions. PROTO = Prototypicality, COMP = competence, SOC = sociability, MOR = morality. Tables from Studies 1a and 2a where occupations are sorted by counts of each study are given on [OSF](https://osf.io/cpjyd/?view_only=9f8ae089d0a34c1e9a8d9ea048121c5f).

**Steps in Study 2a**

1. Manually correct spelling mistakes in Excel (“Physisist” -> “Physicist”)
2. Import database
3. Capitalize first letter (“chemist” -> “Chemist”)
4. Remove spaces before and after (“Chemist “ -> “Chemist”)
5. Remove ‘s’ at the end of occupations (“Chemists” -> “Chemist”)
6. Count the entries (930 unique entries/occupations)
7. Selecting only occupations that have counts over two (190 entries)
8. Renaming occupations:
   1. Psychologist (Psychology)
   2. Pharmacologist (Pharmaceutical)
   3. Chemists (Chemical Scientist, Chemistry, Chemical)
   4. Physicist (Physics)
   5. Geneticist (Genetic)
   6. Environmental Scientist (Environmentalist, Environmental, Environmental Science)
   7. Climatologist (Climate Scientist)
   8. Biologists (Biological, Biology)
   9. Astronomer (Astronomist, Astronomy)
   10. Researcher (Research)
   11. Microbiologist (Micro biologist, Microbiology)
   12. Volcanologist (Vulcanologist)
   13. Food Scientist (Food)
   14. Archeologist (Archaeologist)
   15. Medical Researcher (Medical, Medical Scientist)
   16. Political Scientist (Political, Political Science)
   17. Astrophysicist (Astrophysics, Astro Physicist)
   18. Mathematician (Mathematics)
   19. Geologist (Geology)
   20. Computer Scientist (Computer Science)
   21. Nuclear Scientist (Nuclear)
9. Cut-off (arbitrary in previous studies): 5% i.e., 15 counts. In total, 48 occupations
10. Delete non-scientific occupations (second column represents counts):

| Engineer | 53 |
| --- | --- |
| Doctor | 51 |
| Pharmacist | 50 |
| Astronaut | 30 |
| Physician | 29 |
| Psychiatrist | 19 |
| Veterinarian | 19 |
| Chemical Engineer | 18 |
| Pathologist | 18 |
| Mechanical Engineer | 17 |
| Nurse | 16 |
| Medical Doctor | 15 |

1. Number of occupations: **36**
2. When no pre-processing is done (from step 4 on), a similar list is obtained

**Study 2b - BRM model results**

Before testing for clustering, we again first estimated means from a mixed model which included random intercept for participants (ICC_competence_ = .52, ICC_sociability_ = .52, ICC_morality_ = .58). Ratings for each occupation on BRM dimensions are given in Supplementary Table 1. Means suggest that scientific occupations are highly competent (*M* = 5.97), moderately sociable (*M* = 4.45), and relatively moral (*M* = 5.19). Differences between all dimensions were significant, *t*s > 11.68 p_bonf_ < .001. Correlations between dimensions across occupations are given in Supplementary Table 2.

We used the same approach in determining the number of clusters as in Study 1b. NbClust showed that most indices (10) suggested the 6-cluster solution, which resonates with the findings from the previous study. Both hierarchical clustering algorithm and k-means returned the same cluster memberships. The 3D plot is given [here](https://rpubs.com/vukashg/838572), while the 2D graph which collapsed sociability and morality into one dimension is given in Supplementary Figure 1.

The six-cluster solution from Study 2b replicated the findings from Study 1b, which is why we named clusters in the same way. Again, “scientist” is among the physical and mathematical occupations. Overlap between these and biomedical occupations is even more pronounced in this study, given that “geneticist”, “microbiologist”, and “astronomer” do not belong to their respective content-based clusters. In this study, ecological/wildlife sciences also included “ecologist” (it belonged to unsorted occupations in Study 1b), while “statistician” (new occupation in Study 2b) was classified as data sciences. Interestingly, based on the social evaluations, “psychologist” was classified as an unsorted occupation, which is a cluster with lower perceptions of competence.


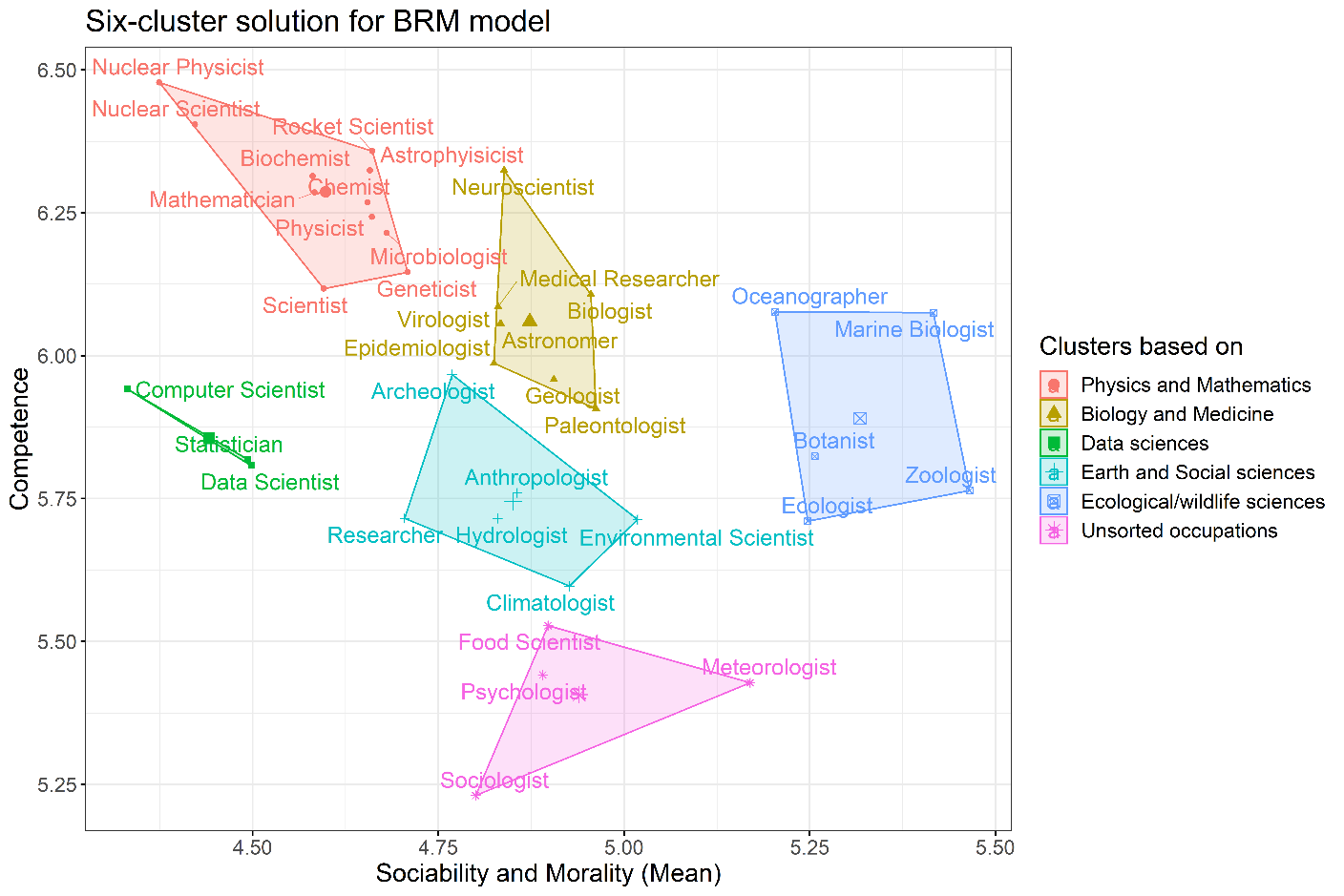


Supplementary Figure 1. Six clusters of scientific occupations based on the ratings of competence, sociability, and morality in Study 2b. For the 2D representation, we collapsed sociability and morality because many models consider them subdimensions of a higher-order factor (named warmth^13^ or communion^19^). Lines represent cluster borders, with each cluster having their own color and symbol. Cluster centroids are represented with a large symbol in the cluster center.

***Social evaluations dimensions (BRM) and Prototypicality***

We next investigated which social dimensions contribute to perceptions of prototypicality. Rating an occupation as more prototypical of scientists was associated with perceptions of higher competence, lower sociability, and higher morality (Supplementary Table 2). Linear regression with three social evaluations as predictors showed that the model was significant, *F*(3,31) = 40.480, *p* <.001, *adj* *R*^2^ = .78. Replicating findings from Study 1b, higher levels of competence contributed to prototypicality ratings (*t* = 5.824, *p* <.001), while sociability and morality did not, (*t* = .921, *p* = .36, and *t* = 1.991, *p* = .055, respectively).

| Supplementary Table 2. Correlations between social perceptions and prototypicality ratings | | | |  |
| --- | --- | --- | --- | --- |
|  | Competence | Sociability | Morality | |
| Competence |  |  |  | |
| Sociability | -.606^***^ |  |  | |
| Morality | .209 | .483^**^ |  | |
| Prototypicality | .817^***^ | -.250 | .508^**^ | |
| *Note*. Correlations with prototypicality did not include generic occupations of “scientist” and “researcher”. ^**^*p* < 0.01^, ***^*p*<.001 | | | |  |

**Study 2b - DPM model results**

***Clusters of occupations***

Before testing for clustering, we first estimated means from a mixed model which included random intercept for participants (ICC_competence_ = .63¸ ICC_assertiveness_ = .65, ICC_warmth_ = .51, ICC_morality_ = .64). Ratings for each occupation on DPM dimensions are given in Supplementary Table 3. Means suggest that, on general, scientific occupations are perceived as highly competent (*M* = 5.99.), relatively assertive (*M* = 5.43) and moral (*M* = 5.39), and moderately warm (*M* = 4.85). Differences between all dimensions except assertiveness and morality (*t* = 1.088, *p* =1.000) were significant, *t*s > 6.791 *p*_bonf_ < .001. Correlation table between all dimensions, and prototypicality is given in Supplementary Table 4.

| Supplementary Table 3. Ratings of warmth, morality, competence, and assertiveness for each occupation | | | | |
| --- | --- | --- | --- | --- |
|  | Warmth | Morality | Assertiveness | Competence |
| Scientist | 4.56 | 5.38 | 5.47 | 6.12 |
| Researcher | 4.73 | 5.34 | 5.47 | 6.00 |
| Chemist | 4.52 | 5.39 | 5.37 | 6.07 |
| Biologist | 4.94 | 5.45 | 5.43 | 6.11 |
| Physicist | 4.51 | 5.35 | 5.50 | 6.25 |
| Geologist | 4.76 | 5.37 | 5.23 | 5.95 |
| Astronomer | 4.73 | 5.46 | 5.49 | 6.15 |
| Marine Biologist | 5.79 | 5.76 | 5.50 | 6.07 |
| Astrophyisicist | 4.37 | 5.26 | 5.51 | 6.16 |
| Psychologist | 5.45 | 5.38 | 5.39 | 5.55 |
| Microbiologist | 4.67 | 5.49 | 5.36 | 6.10 |
| Zoologist | 5.90 | 5.65 | 5.23 | 5.75 |
| Botanist | 5.29 | 5.52 | 5.01 | 5.82 |
| Biochemist | 4.49 | 5.31 | 5.61 | 6.29 |
| Meteorologist | 5.39 | 5.24 | 5.30 | 5.66 |
| Virologist | 4.80 | 5.51 | 5.60 | 6.12 |
| Archeologist | 4.78 | 5.36 | 5.60 | 5.98 |
| Mathematician | 4.33 | 5.36 | 5.44 | 6.31 |
| Environmental Scientist | 5.28 | 5.46 | 5.42 | 5.78 |
| Geneticist | 4.73 | 5.36 | 5.54 | 6.14 |
| Paleontologist | 4.86 | 5.38 | 5.48 | 5.93 |
| Ecologist | 5.41 | 5.62 | 5.50 | 5.89 |
| Medical Researcher | 4.78 | 5.40 | 5.60 | 6.09 |
| Nuclear Physicist | 4.22 | 5.13 | 5.74 | 6.33 |
| Epidemiologist | 5.00 | 5.48 | 5.60 | 6.03 |
| Computer Scientist | 4.29 | 5.10 | 5.08 | 5.84 |
| Data Scientist | 4.38 | 5.25 | 5.22 | 5.98 |
| Food Scientist | 4.98 | 5.34 | 5.14 | 5.68 |
| Rocket Scientist | 4.51 | 5.29 | 5.69 | 6.29 |
| Anthropologist | 5.00 | 5.35 | 5.39 | 5.82 |
| Oceanographer | 5.46 | 5.69 | 5.58 | 6.06 |
| Nuclear Scientist | 4.24 | 5.26 | 5.75 | 6.33 |
| Climatologist | 5.18 | 5.47 | 5.35 | 5.68 |
| Statistician | 4.27 | 5.26 | 5.28 | 5.94 |
| Neuroscientist | 4.75 | 5.45 | 5.69 | 6.19 |
| Sociologist | 5.32 | 5.25 | 5.14 | 5.46 |
| Hydrologist | 4.87 | 5.35 | 5.24 | 5.83 |

| Supplementary Table 4. Correlations between social perceptions and prototypicality ratings | | | | | |
| --- | --- | --- | --- | --- | --- |
|  | Competence | Assertiveness | Warmth | Morality |  |
| Competence |  |  |  |  |  |
| Assertiveness | .717^***^ |  |  |  |  |
| Warmth | -.630^***^ | -.259 |  |  |  |
| Morality | -.047 | .087 | .707^***^ |  |  |
| Prototypicality | .790^***^ | .700^***^ | -.247 | .234 |  |
| *Note*. Correlations with prototypicality did not include generic occupations of “scientist” and “researcher”. ^†^ *p* <.10^, **^*p* < 0.01^, ***^*p*<.001 | | | | | |
